# Treating the “E” in “G × E”: Trauma-Informed Approaches and Psychological Therapy Interventions in Psychosis

**DOI:** 10.3389/fpsyt.2019.00009

**Published:** 2019-01-30

**Authors:** Olympia Gianfrancesco, Vivien J. Bubb, John P. Quinn

**Affiliations:** ^1^Department of Molecular and Clinical Pharmacology, Institute of Translational Medicine, University of Liverpool, Liverpool, United Kingdom; ^2^MRC Human Genetics Unit, Institute of Genetics and Molecular Medicine, University of Edinburgh, Edinburgh, United Kingdom

**Keywords:** psychosis, schizophrenia, trauma, stress, psychological therapy, epigenetics, gene × environment

## Abstract

Despite advances in genetic research, causal variants affecting risk for schizophrenia remain poorly characterized, and the top 108 loci identified through genome-wide association studies (GWAS) explain only 3.4% of variance in risk profiles. Such work is defining the highly complex nature of this condition, with omnigenic models of schizophrenia suggesting that gene regulatory networks are sufficiently interconnected such that altered expression of any “peripheral” gene in a relevant cell type has the capacity to indirectly modulate the expression of “core” schizophrenia-associated genes. This wealth of associated genes with small effect sizes makes identifying new druggable targets difficult, and current pharmacological treatments for schizophrenia can involve serious side effects. However, the fact that the majority of schizophrenia genome-wide associated variants fall within non-coding DNA is suggestive of their potential to modulate gene regulation. This would be consistent with risks that can be mediated in a “gene × environment” (G × E) manner. Stress and trauma can alter the regulation of key brain-related pathways over the lifetime of an individual, including modulation of brain development, and neurochemistry in the adult. Recent studies demonstrate a significant overlap between psychotic symptoms and trauma, ranging from prior trauma contributing to psychosis, as well as trauma in response to the experience of psychosis itself or in response to treatment. Given the known effects of trauma on both CNS gene expression and severity of psychosis symptoms, it may be that pharmacological treatment alone risks leaving individuals with a highly stressful and unresolved environmental component that continues to act in a “G × E” manner, with the likelihood that this would negatively impact recovery and relapse risk. This review aims to cover the recent advances elucidating the complex genetic architecture of schizophrenia, as well as the long-term effects of early life trauma on brain function and future mental health risk. Further, the evidence demonstrating the role of ongoing responses to trauma or heightened stress sensitivity, and their impact on the course of illness and recovery, is presented. Finally, the need for trauma-informed approaches and psychological therapy-based interventions is discussed, and a brief overview of the evidence to determine their utility is presented.

## Introduction

Early studies aimed at identifying underlying genetic components in schizophrenia highlighted both rare disruptions and common variants in a number of candidate genes such as Disrupted In Schizophrenia 1 (*DISC1*) (1, 2). These variants were identified in individuals with a diagnosis of schizophrenia and other major mental health conditions through pedigree analysis, though many schizophrenia candidate gene studies from this era suffered from lack of statistical power and reproducibility (3). Recent genome-wide association studies (GWAS) have significantly advanced our understanding of the genetic architecture of schizophrenia, revealing that risk for this condition is highly polygenic in nature (4). Comparing GWAS data on historical candidate gene variants did not provide supporting evidence for enrichment of GWAS signal at many of these previously identified loci (3, 5, 6), suggesting that implicated variants from early studies may not be representative of risk in the majority of individuals with this condition.

Schizophrenia GWAS studies have since identified 108 highly associated loci (4), and have provided some support for the utility of commonly used antipsychotic medications by highlighting variants at dopamine receptor genes, such as *DRD2*, to be associated with schizophrenia (7). However, currently available antipsychotic treatments can cause serious side effects in a number of individuals, such as increasing risk for metabolic and cardiovascular conditions (8–11).

Druggable target analysis of GWAS data has suggested a potential role for the repurposing of epilepsy medications and calcium channel blockers as treatment options to be explored (7). However, a recent omnigenic model of schizophrenia suggested that all expressed genes in a relevant cell type may have an effect on risk (12), and as such, the large number of implicated genes with small effect sizes, and the broad distribution of risk variants across the genome (13), has hindered the identification of new druggable targets.

That the large majority of schizophrenia GWAS single nucleotide polymorphisms (SNPs) fall in non-coding DNA (4), with enrichment in open chromatin and predicted transcriptional regulatory elements (12, 14, 15), would suggest that risk for this condition is likely to be mediated through gene-environment (G × E) interactions (16). Indeed, while polygenic risk scores (PRS) have typically been associated with increased odds for schizophrenia (4), a recent study examining polygenic risk with or without obstetric complications suggested that risk may be highly modified by the effect of early life stressors (17). Evidence supporting the effect of early life stress and trauma on psychosis risk is robust (18, 19), with an evident dose-response relationship between cumulative trauma exposure and increasing odds for experiencing psychosis (18, 20, 21). Similarly, studies have demonstrated that exposure to specific types of maltreatment in childhood can increase the odds of experiencing specific psychotic symptoms (21).

One in three cases of psychosis are predicted to be due to the effects of childhood trauma (18, 19), with complementary evidence demonstrating the beneficial and protective epigenetic effects of positive parental nurturing in early infancy and childhood (22, 23). As such, early experiences of trauma are one of the most preventable risk factors for psychosis and schizophrenia. Individuals with a history of psychosis or psychosis-like experiences are significantly more likely to have experienced trauma than those without a history of such experiences (24–26). Exposure to early trauma has been suggested to modulate developmental and molecular pathways such that an individual may become sensitized to future life stress (27–29). Indeed, high stress sensitivity has been correlated with increased rates of psychosis-like symptoms in the general population across 39 countries (30), and with significantly increased symptom intensity in individuals with first episode psychosis (FEP) compared to controls (31). In individuals with experiences of psychosis, exposure to childhood maltreatment has also been associated with significant increases in emotional and psychotic reactivity to small daily life stressors in adulthood (32). Such research would support the hypothesis that early life stress can predispose individuals to greater stress reactivity in adulthood, which would modulate risk for experiencing psychotic symptoms.

In addition to trauma as a risk factor for psychosis, there is a growing recognition that the experience of psychosis itself, and the distress associated with treatment methods, can result in new or further traumatization. Up to 69% of individuals that have experienced psychosis suffer post-psychosis trauma symptoms, while up to 39% develop clinical post-traumatic stress disorder (PTSD) in response to the events of their psychotic episodes, including distressing treatment-related events such as involuntary hospitalization (33–37). It is therefore clear that a significant subset of individuals with psychosis have endured repeated trauma, and may be re-traumatized during the course of illness and treatment (38, 39). Such experiences are likely to negatively impact treatment response and recovery. For example, individuals with “treatment resistant” schizophrenia report exposure to significantly higher rates of stressful life events and childhood trauma than those with medication-responsive symptoms (40, 41).

Stressful experiences can modulate the regulation of central nervous system (CNS) genes and genes involved in stress response (42–44), though preliminary studies have demonstrated that psychological therapy can normalize epigenetic marks over key CNS genes (45–47), suggesting that non-pharmaceutical treatments are also a plausible route to biological intervention in the context of mental health. Given this information, research into the provision and utility of psychological therapy for individuals with psychosis is currently being undertaken. Trauma-informed psychological interventions have proven safe and acceptable for use with those experiencing psychosis (48–50), and have been shown to be effective in reducing post-psychosis trauma (51). Similarly, Cognitive Behavioral Therapy (CBT) has been adapted for use with individuals experiencing psychosis (CBTp) and has proven to be both safe and effective for use in this population (52, 53), such that national guidelines in the UK now recommend this as a frontline treatment for those experiencing psychosis, or those at high risk (54). CBT for individuals with psychosis has been shown to result in sustained improvements in positive symptoms (55–58), as well as improvements in depression, anxiety, quality of life, and functional outcomes, which were maintained at follow up (57, 58). Sustainable improvements in both positive and general psychosis symptoms have also been demonstrated in a trial of CBTp with individuals whose condition had previously been classified as medication-resistant (55).

This review aims to provide a brief outline of the genetic complexity of schizophrenia, the impact of trauma on brain development, function, and future mental health, and both the need and evidence base for trauma-informed approaches and psychological therapy-based interventions.

## The Current Landscape of Schizophrenia Genetics Research

### Insights From Schizophrenia GWAS

The most recent advances in schizophrenia genetic research have come from large-scale GWAS, which have identified many common SNPs with small effect sizes, allowing the calculation of polygenic risk scores (PRS) based on hundreds of low effect alleles. The largest schizophrenia GWAS to date identified 128 variants at 108 genomic loci (4). However, these risk SNPs still only identify a very small percentage of risk, and the frequency of risk-associated alleles typically differ by <2% between case and control cohorts (59). The large majority of risk SNPs identified through GWAS reside in non-coding regions, which suggests that much of the identified genetic risk may be highlighting gene regulatory mechanisms. However, it is important to note that many such associated SNPs may have only reached significance as a consequence of being in linkage disequilibrium (LD) with truly causal SNPs, while conferring no risk-related functional alteration on their own. Despite this, a number of studies have confirmed that individual GWAS SNPs at key schizophrenia-associated loci reside in regulatory regions that are active in the CNS (60, 61), and that have functional consequences based on SNP genotype (62, 63).

It has previously been assumed that genetic variants that are associated with disease risk would be clustered at disease relevant gene loci, or around genes with relevant tissue-specific expression. However, it has been argued that enrichment of GWAS signal at specific disease relevant loci is weak for many complex traits and conditions (12). In the case of schizophrenia, work by Loh et al. demonstrated that 71–100% of all 1 Mb windows in the human genome contain one or more identified GWAS variant(s) which would influence schizophrenia risk (13). However, within such blocks in the cortex, Bryois et al. found that schizophrenia-associated SNPs were enriched in regions of open chromatin (15). Such findings provide further evidence consistent with a G × E model, in which our environment is likely able to modulate our neurochemistry, with potential effects on our risk for mental health conditions (16).

As stated, GWAS signal was found to be strongly enriched in regions of chromatin that are active in disease-relevant cell types. However, work by Boyle et al. demonstrated that, while genes with brain-specific expression patterns were more strongly enriched for schizophrenia-associated GWAS SNPs, SNPs within or near to genes with broad and non-specific expression profiles contributed more to the total schizophrenia heritability due to their larger number (12). Boyle et al. also noted weak enrichment for relevant functional ontologies associated with identified schizophrenia risk loci, which would be consistent with results from the Psychiatric Genomics Consortium (4).

Such findings have resulted in a hypothesized “omnigenic model” of complex conditions such as schizophrenia, in which a small number of variants have regulatory effects on core pathways central to schizophrenia, while a much larger number of variants influence expression of a peripheral gene set which can modify the action of the core gene set. This model would support the finding that variation affecting genes in core associated pathways explains only a small fraction of schizophrenia heritability, while the remaining heritability may be explained by many small variations across all other expressed genes (4, 12).

### Uncovering Functional Mechanisms of GWAS Variants

Current research is beginning to integrate GWAS data with expression and methylation quantitative trait loci (eQTLs, meQTLs) to determine patterns of gene expression and altered regulation that may contribute to schizophrenia biology (64–67). Similar work is beginning to overlay GWAS data with chromatin profiles in relevant cell types in order to identify potential chromatin-modifying effects of GWAS SNPs.

Environmental stressors, such as childhood adversity, have been associated with altered methylation across CNS-expressed genes, such as FK506 binding protein 5 (*FKBP5*) and glucocorticoid receptors, which are known regulators of the stress response and have been associated with psychiatric conditions such as depression and PTSD (42–44). Further, methylation of the KIT ligand (*KITLG*) locus, which encodes the ligand for the receptor tyrosine kinase, KIT, has been shown to mediate the association between childhood trauma and cortisol stress response, and has been highlighted as a region of interest with regard to epigenetic programming of stress reactivity (68).

Multiple different types of early life trauma have been demonstrated to alter methylation at the glucocorticoid receptor in peripheral blood of individuals with diagnosed psychiatric conditions (42). Those that had been exposed to sexual abuse, physical abuse/neglect, and emotional abuse/neglect, had significantly higher levels of methylation across multiple CpG regions at the glucocorticoid receptor locus compared to those without such exposures. Further, a dose-response relationship was evident, in which exposure to multiple trauma types, or increased trauma severity, was associated with higher levels of methylation at this region (42). Methylation changes in response to trauma can also be mediated in a genotype-dependent manner, as in the case of *FKBP5* (43).

Schizophrenia GWAS SNPs are known to significantly overlap with eQTLs and meQTLs in the adult hippocampus, frontal cortex, and the fetal brain (69–71), suggesting that some associated variants may mediate risk by influencing gene methylation, potentially in a differential manner in response to environmental stimuli. Fetal brain meQTLs have been found to be enriched in functional elements and were similarly demonstrated to be over-represented for schizophrenia GWAS variants and at schizophrenia-associated genomic loci (69). The majority of fetal brain meQTLs (83.46%) showed evidence of functionality in at least one of the adult brain regions tested in this study (pre-frontal cortex, striatum, and cerebellum), though such shared fetal and adult meQTLs that were enriched at schizophrenia-associated loci were found to have stronger effects in the fetal brain than in the adult brain. The remaining subset of fetal-specific meQTLs were also found to be enriched at genomic loci that had previously been associated with schizophrenia through GWAS (69). That many fetal meQTLs in the brain are preserved in adulthood may suggest a mechanism through which the effects of early environmental challenges can persist, influencing epigenetic risk and neurodevelopment in ways which may modulate the likelihood of experiencing psychiatric conditions (64).

Similarly, ATAC-seq (Assay for Transposable Accessible Chromatin) on post-mortem dorsolateral prefrontal cortex (DL-PFC) samples from 135 adults with a diagnosis of schizophrenia and 137 controls demonstrated that schizophrenia GWAS SNPs were enriched in accessible chromatin regions in the brain, with 1.2% of the GWAS SNPs in DL-PFC ATAC-seq peaks accounting for 8.55% of schizophrenia SNP heritability (15). ATAC-seq peaks from fetal brains were also enriched for schizophrenia association, demonstrating that such risk-related regulatory loci are accessible, and thus likely active, during early development (15). Taken together, such findings may provide a mechanism through which neurodevelopmental risk factors, such as maternal stress or immune activation, may have a lasting impact on the developing and adult brain by affecting gene expression through the modulation of methylation and chromatin accessibility.

### Insights and Complications in Using Polygenic Risk Scores

Additional utility for the wealth of GWAS data has been found in the generation of PRS. PRS use data on variants across multiple associated loci in order to try to predict risk for a particular condition based on combined SNP genotypes. To date, multiple studies have demonstrated that increased polygenic risk for schizophrenia is significantly associated with increased odds ratios (OR) for schizophrenia-spectrum diagnoses, such as schizophrenia, schizoaffective disorder, first episode psychosis and psychosis not otherwise specified (4, 72, 73). The odds ratio is a measure of association between an exposure and an outcome. In this instance, increased overall genetic risk for schizophrenia is associated with a higher likelihood of schizophrenia-spectrum diagnosis. Notably, the landmark publication on recent schizophrenia genetics research identifying 108 schizophrenia-associated loci demonstrated that the highest polygenic risk scores in their analysis were associated with an odds ratio for schizophrenia of 7.8–20.3 (4). However, almost all studies on PRS and schizophrenia risk did not account for the influence or interaction with other known risk factors, such as early life stress. Further work has now begun examining to which risk-related traits the PRS may predispose, and how polygenic risk may interact with other such risk factors.

Higher schizophrenia PRS have been associated with increased levels of anxiety in a non-clinical adolescent population, but failed to demonstrate an association with psychotic experiences (74–76). This may suggest that schizophrenia risk in adolescence presents as, or contributes to, known phenotypes that are associated with schizophrenia in adulthood, such as anxiety or increased stress sensitivity. Higher schizophrenia PRS have also been associated with reduced hippocampal volume in FEP individuals and those classified as having an “at risk mental state” (ARMS) (73), as well as with multiple immune conditions (77), which provides supporting evidence for immune system mechanisms in schizophrenia.

The interaction of schizophrenia GWAS SNPs with environmental stressors, including early life complications (ELCs) such as complications during pregnancy, labor, and delivery, have further been shown to influence the effects of schizophrenia PRS (17). Ursini et al. constructed schizophrenia PRS using GWAS SNPs from meta-analyses of Psychiatric Genomics Consortium (PGC) data sets (17). Odds ratios for schizophrenia were calculated for each subset of PRS in comparison to the lowest polygenic risk quintile, in individuals with or without ELCs. For individuals in the highest polygenic risk quintile without ELCs, no statistically significant change in odds ratio for schizophrenia was observed. These findings were validated in separate Italian and German case-control cohorts, with the finding that five times more schizophrenia risk was explained by an individual's PRS in the presence of ELCs compared to such scores in the absence of ELCs. This relationship was seen for polygenic risk scores constructed using statistically significant GWAS SNPs, and also for PRS constructed with SNPs showing a trend toward genome-wide significance, but not for other putatively associated SNPs. The authors suggested the possibility that the high statistical significance of these SNPs identified by GWAS in a heterogeneous group of case samples may be due to their interaction with common environmental risk factors, which, in combination, would confer a strong effect on risk (17). Such work may in part explain the relatively low penetrance of common schizophrenia risk variants when assessed or inherited without the appropriate environmental context through which they exert their influence on mental health risk.

A study by Curtis further demonstrated that, while PRS were significantly associated with schizophrenia in the Common Mind Consortium data set, these measures were not robustly associated with expression of any of the 16,423 individual genes tested, nor with expression of specific schizophrenia-associated gene sets (78). Curtis suggested that common variants may instead be influencing indirect risk factors that may be far removed from core biological pathways, such as increasing risk for, or response to, schizophrenia-associated environmental factors. Indeed, Ursini et al. found that PRS for schizophrenia increased the likelihood of experiencing an ELC (17), and further PRS have been shown to significantly predict risk for multiple immune conditions (77). This could provide support for the hypothesis that polygenic risk for schizophrenia may be identifying predisposition to other environmental risk factors for this condition (78), such obstetric complications, or altered immune function that may increase the likelihood of maternal infection.

### Schizophrenia Risk Is Likely to be Driven by Environmental Challenge

Recent insights into the genomic underpinnings of schizophrenia have been successful in more clearly elucidating the complex polygenic architecture of this condition, and in providing support for the biological targets of currently available pharmacological treatments. However, schizophrenia GWAS signal is widely distributed across the genome, and does not necessarily converge on particular loci or novel molecular pathways (13). This has hindered the identification of new targets for improved pharmacological intervention. While PRS for schizophrenia have for the most part shown reliable increases in risk with increased genetic burden, emerging research in this area is beginning to highlight roles for environmental interaction with genetic risk, while raising the possibility that PRS may in part be predisposing to other schizophrenia-associated risk factors (e.g., obstetric complications) rather than the condition itself (17, 78). This may suggest that research should target a better understanding of these risk mechanisms, by which environmental stressors modulate biological pathways to explain observed alterations in schizophrenia.

## Early Life Stress and Schizophrenia Risk

Polygenic risk scores in schizophrenia point toward a potential for genetic risk to be mediated through environmental stress (17, 78). This section examines the evidence for one of the most significant and preventable risks for psychosis and schizophrenia (18, 19), the experience of early life stress and trauma, the mechanisms through which this may increase risk, and potential treatment options.

The experience of trauma can be defined as a highly stressful event that involves serious threat to one's physical wellbeing and to one's sense of self, as well as overwhelming one's capacity for coping (79). The Diagnostic and Statistical Manual of Mental Disorders 5th Edition (DSM-V) defines a traumatic event as involving actual or perceived threat of death, serious injury, or violence (80). Such experiences are likely to involve intense feelings of fear and helplessness (81), though these criteria are no longer required to define a traumatic experience in the DSM-V. Further, traumatic experiences can be encountered in a number of ways, including direct personal exposure to trauma, witnessing another person's experience of trauma, or in indirect ways such as coping with the trauma experience of a close associate, or repeatedly being exposed to the consequences of traumatic events in a professional role (such as emergency responders, or investigators of violent crime).

It is important to note that the studies covered in this review largely consider only experiences of direct personal trauma, and of trauma that is of an interpersonal nature, for the most part involving victimization. This includes experiences of multiple types of abuse (emotional, physical, sexual) and neglect, as well as experiences such as bullying. Other types of trauma experiences, such as natural disasters or sudden, life-threatening illnesses, were not included in this review. Further, the effects of exposure to pre- or peri-natal stressors are not covered in this review, nor are the effects of early stress that may be linked to poverty or other social factors.

### Early Life Stress as a Risk Factor for Psychosis and Schizophrenia

Psychologically healthy individuals who report psychosis-like experiences have been found to have higher incidences of childhood trauma (24–26). Early experiences of trauma and adversity are well known risk factors for mental illness, with a World Health Organization (WHO) study of almost 52,000 individuals across 21 countries demonstrating that childhood adversity accounted for 30% of all adult mental health conditions (82). Similarly, a meta-analysis of studies assessing the link between childhood adversity and psychosis demonstrated that 78% of studies tested showed a positive association between childhood adversity and psychosis, and that 33% of the population risk for psychosis could be attributed to experiences of childhood adversity (18). A more recent study of 23,998 individuals examining the population attributable risk of childhood adversity on psychosis demonstrated similar results, finding 31% of psychosis cases to be attributable to early adversity (19).

Individuals at clinical high risk (CHR) for psychosis typically also report higher rates of childhood trauma. A meta-analysis of six studies on ultra-high risk (UHR) populations demonstrated a significant increase in exposure to childhood trauma (83). Loewy et al. further demonstrated that exposure to trauma is typically experienced in early life (before age 12) in clinical high risk populations, and occurs prior to the onset of clinical high risk symptoms (84).

Studies to assess the reliability of retrospective trauma reporting in individuals experiencing psychosis demonstrated that reports of trauma are consistent across time and are not significantly affected by current mental health status (85). Fisher et al. also demonstrated that retrospective trauma reporting was consistent with independent clinical case notes from abuse reports (85). However, a number of studies have also reported that minimization and denial of trauma is common in both case and control populations (86, 87), with minimization and denial of trauma being significantly increased in controls compared to individuals with psychosis in a recent study by Church et al. Despite this, individuals with experiences of psychosis still had significantly higher rates of self-reported childhood trauma (61% of case individuals reporting trauma, compared to 15% of controls) after correcting for minimization and denial scores in this study (87). Indeed, MacDonald et al. have suggested that current estimates of the relationship between childhood trauma and psychiatric conditions may be conservative due to the under-reporting of trauma experiences. The authors further suggest that future studies investigating childhood trauma should measure minimization and denial scores in order to account for such biases (86).

While specific types of adverse experience have all been shown to increase overall psychosis risk (18), it has further been demonstrated that specific types of adversity show a correlation with specific psychotic symptoms (21, 88). For example, Bentall et al. demonstrated that childhood rape was associated with a significantly increased risk of hearing voices, but did not significantly alter risk of paranoia (21). On the other hand, growing up in institutional care was significantly associated with increased odds of experiencing paranoia, but did not significantly alter risk of voice hearing. Some incidences of trauma were also demonstrated to significantly increase the risk of both voice hearing and paranoia, such as in the case of physical abuse (21).

Furthermore, experience of trauma has been shown to have a cumulative, dose-response effect on psychosis risk (18–21, 89). For example, Shevlin et al. demonstrated that experiencing two or more traumas (in childhood and/or adulthood) was predictive of psychosis, with increased number of traumas also demonstrating an additive effect on risk (20). Similarly, the 2012 meta-analysis by Varese et al. highlighted that nine of the 10 studies that tested for dose response in their analysis demonstrated a positive correlation between increased number of adverse events and increased risk of psychosis (18). This pattern also remained when considering symptoms individually rather than diagnosis. Bentall et al. demonstrated the dose response effect of trauma for risk of paranoia and hearing voices, with a single adverse experience significantly increasing the risk for both, and additional traumatic experiences having an additive effect on risk (21).

A recent meta-analysis of 29 studies into the effect of childhood trauma on symptom severity in individuals with psychosis demonstrated that childhood trauma was significantly associated with increased severity of hallucinations and delusions (90). On the other hand, childhood neglect was significantly associated with increased negative symptoms and hallucinations (90). Total trauma scores, as well as sexual abuse, were significantly associated with increases in hallucinations, while total trauma and sexual abuse (but not neglect) were associated with increased delusions (90). Such work suggests that, as well as predisposing to psychosis, individuals with experiences of childhood trauma are also likely to experience more severe psychotic symptoms.

### Stress Sensitivity and Sensitization as a Mechanism Mediating the Association of Trauma and Psychosis

Stress sensitivity is known to be correlated with the incidence of psychotic experiences (30). An individuals' stress response may be genetically influenced, but is also known to be modified by previous experience, in which early life stress can sensitize an individual to future stress (91, 92). Using World Health Survey data across 39 countries and 176,934 individuals, DeVylder et al. ranked individuals based on stress sensitivity into groups from 2 to 10 (least to most stress sensitive). Each increasing rank in stress sensitivity was associated with incremental increases in the prevalence of psychotic experiences, with stress sensitivity significantly increasing the odds of an individual reporting psychotic experiences (30). This association remained unchanged when carrying out the analysis either including or excluding those with a self-reported diagnosis of psychosis, and when separating the analyses for hallucinations and delusions. Such data would suggest that increased stress sensitivity is associated with increased risk of experiencing psychotic symptoms; both in the general population and in those with diagnosed psychotic conditions, and that this relationship is shared throughout many countries and cultures across the world (30).

As well as increasing risk, stress sensitivity has further been associated with the intensity of psychotic experiences in both case and control populations. For example, Reininghaus et al. demonstrated that increased sensitivity to multiple types of stressful situations was significantly associated with intensity of psychotic experiences in FEP individuals, ARMS individuals, and in healthy controls (31). When comparing groups, increased event-, activity-, and area-stress sensitivities were significantly associated with increased intensity of psychotic experiences in FEP individuals compared to controls.

**Figure 1:**
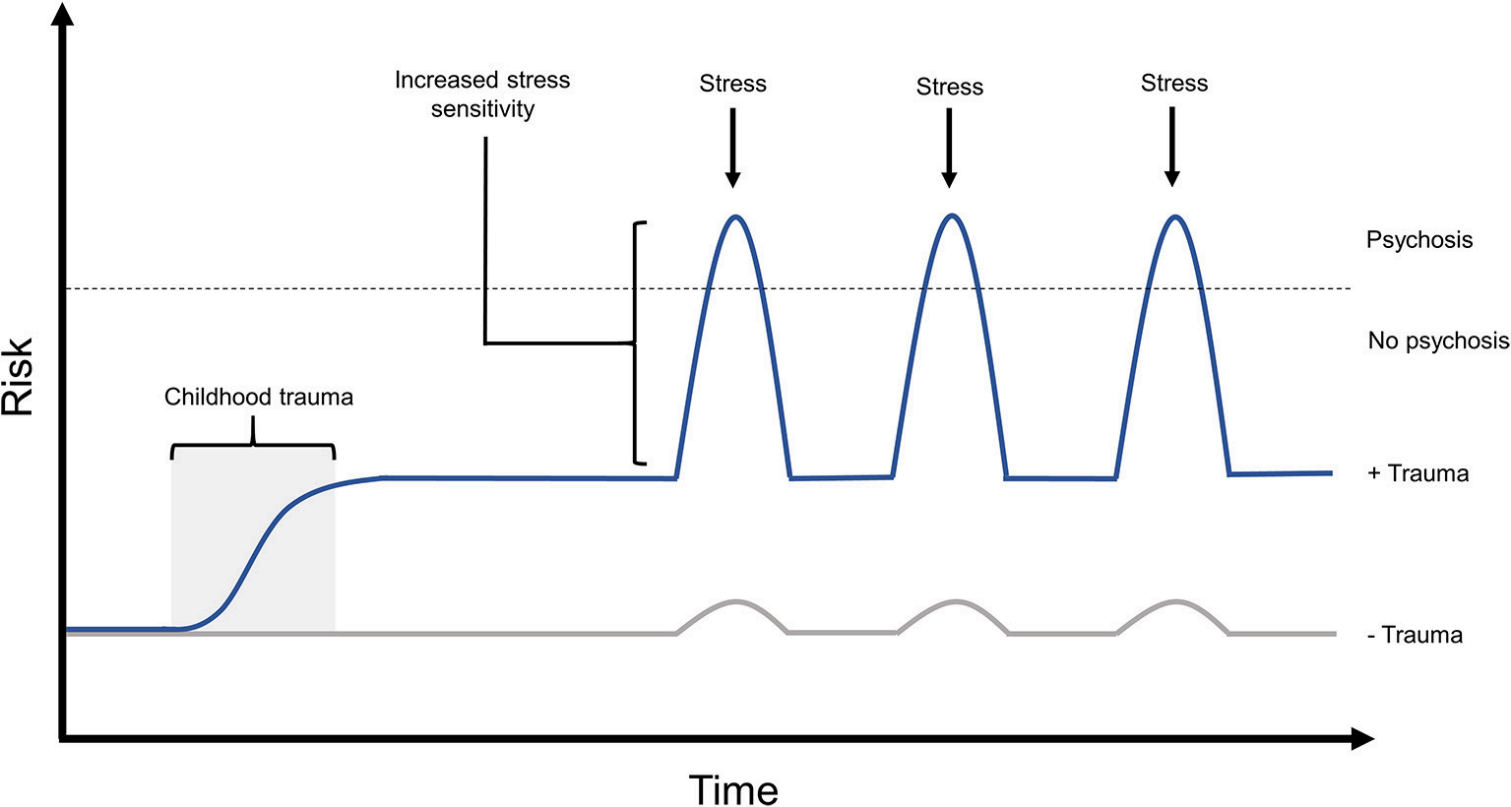
The effects of childhood trauma on stress sensitivity and psychosis risk. Exposure to childhood trauma has been shown to be strongly associated with risk of experiencing psychosis. Such early adversity has also been demonstrated to increase stress sensitivity, both in non-clinical populations and in those experiencing psychosis, which is known to increase both risk and intensity of psychotic experiences. In this model, experience of early trauma is predicted to increase an individual's baseline risk for psychosis, while predisposing them to react more strongly to future stressors. This would further amplify psychosis risk when faced with later stressful experiences during adolescence or adulthood. Risk in this model can also be modulated by the individual's genotype, and allele-specific responses to environmental stressors, the effects of which are not shown in this diagram.

In individuals with experiences of psychosis, exposure to childhood trauma was correlated with increased emotional and psychotic reactivity to small daily life stressors in adulthood, suggesting that early stress can sensitize individuals to future stress in a way that may modulate their risk for psychotic experiences, and may modulate the intensity of such psychotic experiences (32) (Figure 1). Supporting this finding, there is a known dose-response relationship between increased childhood adversity and more significant increases in daily life stress sensitivity in adulthood (93). Such work suggests a process of sensitization to stress, in which those exposed to early adversity may be sensitized to react more strongly in future to daily stressors. This sensitization process may then modulate risk for experiencing psychosis, or in individuals already experiencing psychosis, may contribute to increased severity of these experiences.

Cristóbal-Narváez et al. have provided evidence of this mechanism specifically in psychosis-like experiences, in a cohort of non-clinical adolescents (92). In this study, experiences of abuse and neglect were associated with psychotic-like and paranoid experiences, while exposure to bullying was associated only with psychotic-like experiences. Abuse, bullying, and neglect were significantly associated with negative affect. All types of childhood adversity tested were found to be associated with daily life stress reactivity, and adversity of an interpersonal nature (bullying, abuse, neglect, and loss) mediated the psychotic- and paranoid-like response to day-to-day social and situational stressors. In particular, individuals reporting higher exposure to bullying were shown to experience significantly higher levels of paranoia in response to social stress than those with lower rates of bullying (92).

### The Effects of Early Trauma on Brain Structure and Function

**Figure 2:**
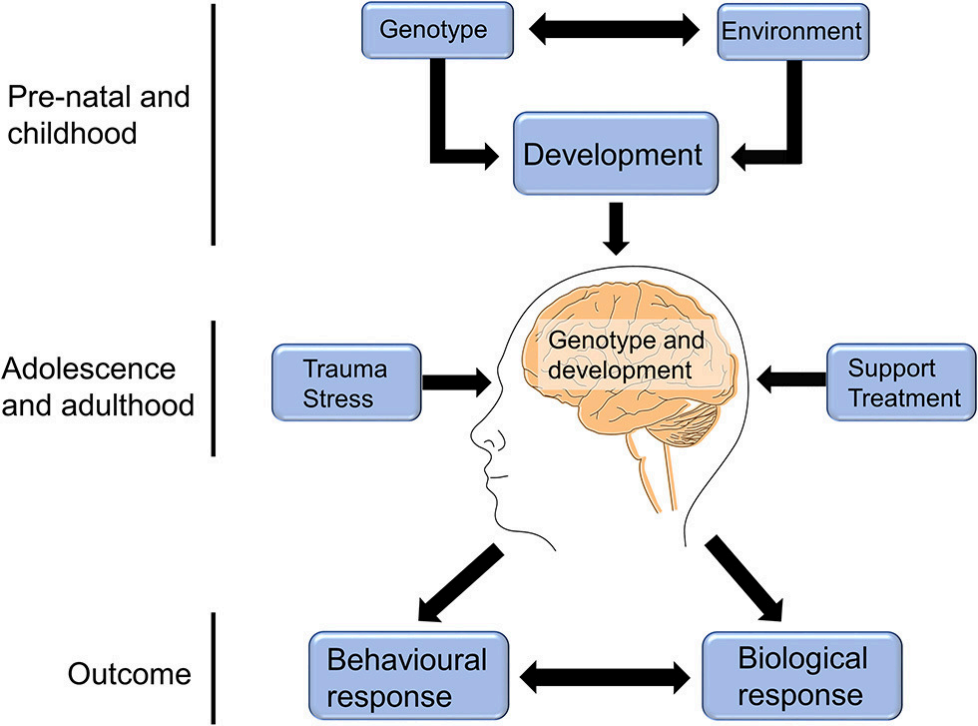
The traumagenic neurodevelopmental model of psychosis. The traumagenic neurodevelopmental model of psychosis suggests that the interaction between an individual's genotype and environment would influence brain development in ways that could modulate psychosis risk in response to future life stress. For example, childhood trauma may negatively impact a child's brain development, resulting in molecular or structural changes that affect their response to adversity later in life. Experiences in adolescence and adulthood can then either result in further increased risk, such as in the case of chronic stress or further trauma experiences, or can reduce the individual's risk, in the case of receiving social support or appropriate treatment. The combination of genotype, brain development, and adolescent/adult life experiences can combine to influence the individual's molecular and biological response, as well as their behavioral response to future stress, which can in turn interact with and impact their risk of experiencing psychosis. This figure is reprinted, with alterations, from Heim et al. (28).

Childhood adversity was found to be associated with changes in brain structure and connectivity in non-clinical adolescent and adult groups (91, 94, 95), with such physical changes being shown to correlate with mental health-related traits that were likely to sensitize the individual to future life stress (91, 94). The traumagenic neurodevelopmental model of psychosis (Figure 2) posits that experiences of early life stress, combined with genetic risk, have the potential to alter the trajectory of brain development or neurochemistry in ways that may put an individual at higher risk of experiencing psychosis in response to future adversity (27, 29).

Studies have demonstrated the association between childhood trauma and alterations in brain structure and connectivity (95, 96). At present, a number of studies have further demonstrated that these trauma-associated structural changes result in increased stress sensitivity in adulthood (91, 94). For example, Gorka et al. demonstrated that childhood adversity was correlated with differences in hippocampal and medial prefrontal cortex gray matter volume in a sample of non-clinical individuals (91). Increasing childhood trauma scores were significantly associated with decreased gray matter volume in the left hippocampus and the medial prefrontal cortex (mPFC), but showed no association with gray matter volume in the right hippocampus or amygdala. Both childhood adversity and decreased gray matter were also correlated with increased anxiety in response to recent life stress. They further demonstrated that the association between decreased hippocampal and mPFC gray matter volume and increased anxiety in response to current stress was significantly mediated by trauma exposure, suggesting that early stress-induced alterations in brain structure can result in increased stress sensitivity in adulthood (91).

Similar studies in adolescents by Herringa et al. have shown that increased childhood maltreatment scores are predictive of lower resting state functional connectivity (rs-FC) between the hippocampus and subgenual cingulate in both male and female adolescents, and of lower rs-FC between the amygdala and subgenual cingulate in female adolescents (94). The effect of childhood maltreatment on the connectivity of these regions remained significant after correction for current life stress. Lower amygdala- and hippocampus-subgenual cingulate connectivity, as well as higher childhood maltreatment scores, was found to be predictive of higher rates of internalizing symptoms (found in depression and anxiety) in adolescents. In both male and female adolescents, experience of childhood maltreatment predicted lower total rs-FC, which in turn modulated the relationship between childhood maltreatment and internalizing symptoms (94). This work provides evidence for the hypothesis that childhood trauma can modulate brain connectivity, resulting in increased levels of internalizing symptoms in a non-clinical adolescent population. Evidence was not found to support reversal of this model (i.e., childhood trauma leads to internalizing symptoms, which results in altered connectivity), thereby suggesting that the effect of trauma on brain connectivity increases internalizing behavior, rather than such behavior affecting brain connectivity.

A recent review on this subject summarizes the evidence suggesting specific effects of different childhood trauma types on situation-relevant brain regions (97), such as alterations in auditory and language processing regions in those who were exposed to repeated verbal abuse (98, 99), and alterations in brain regions associated with processing sensory information from the genitals in those who had experienced sexual trauma (100). While such findings require replication, the authors suggest an evolutionary hypothesis in which these CNS alterations in response to trauma may be viewed as adaptive mechanisms to facilitate survival in a threatening environment (97).

In line with the traumagenic neurodevelopmental model, and adding another layer of complexity to this hypothesis, an individual's genotype has been shown to modulate the effects of childhood trauma on changes in brain structure. For example, Dannlowski et al. have demonstrated the effect of the oxytocin receptor SNP, rs53576, on ventral striatum gray matter volume in individuals with varying levels of childhood trauma (101). Individuals who were homozygous for the G allele of rs53576 showed marked decreases in ventral striatum gray matter volume in response to childhood trauma, compared to A allele carriers, who showed a small increase in gray matter volume at this region in individuals with the highest trauma scores (101).

Similarly, Morey et al. demonstrated a significant interaction between the genotype of the intergenic SNP, rs9373240, and childhood trauma in influencing caudate volume in military service members with experiences of childhood trauma (102). In individuals with no childhood trauma, the homozygous T rs9373240 genotype was not associated with caudate volume. However, in those reporting experiences of early trauma, rs9373240 TT individuals demonstrated significant association with caudate volume in a dose-dependent manner. Those reporting one type of trauma exposure showed significant association of SNP genotype with caudate volume, while those with experiences of two or more types of trauma showed much more significant association of rs9373240 genotype with caudate volume (102). The TT genotype of rs9373240 was significantly associated with increased caudate volume in individuals with experiences of childhood trauma exposure, but was not associated with PTSD diagnosis in this cohort.

Given the above studies, it is clear that early adversity is a substantial and preventable risk factor for psychosis, and that a significant proportion of individuals experiencing psychosis are likely to have adapted both behaviorally and biologically to respond differently to stress as a result of surviving early threatening experiences. Recent research demonstrates that increased stress sensitivity is a mechanism influencing psychosis risk and symptom severity (30, 31), and as such is likely to offer an additional route to intervention. For this reason, interventions such as psychological therapy, which aim to understand the impact of our life experiences on our behavior and current difficulties, and assist the individual in coping with present life stressors, have been gaining traction in the field of psychosis research and treatment. The following section considers the effects of stress and trauma on recovery, and the suitability of psychological interventions for individuals with experiences of psychosis.

## Toward Trauma-Informed Treatment Options for Individuals With Psychosis

Exposure to early life stress is known to influence symptom severity and outcomes with regard to pharmacological treatment with antipsychotic medications. The severity of early life trauma has been shown to be significantly correlated with symptom severity in individuals with psychosis (90). Similarly, those with “treatment resistant” schizophrenia displayed significantly higher levels of stressful life events, and scored more highly on measures of emotional trauma and general trauma, than those that respond to pharmacological treatment (40, 41). There is also suggestive evidence that childhood trauma can affect response to antipsychotic treatment in a genotype dependent manner. For example, multiple SNPs across the matrix metallopeptidase 9 (*MMP9*) gene have been implicated in antipsychotic treatment response, as measured by percentage change in Positive and Negative Symptoms Scale score (PANSS) (103). McGregor et al. found that the PANSS score change associated with SNP alleles in *MMP9* was modulated by exposure to childhood trauma. In particular, homozygotes for the A allele of rs13925 without trauma presented as significant responders to antipsychotic treatment, with a 10.5% reduction in PANSS scores compared to AG or GG individuals. However, individuals with the AA genotype that had experienced early life stress presented as non-responders to treatment, with an increase of 1.67% on the PANSS scale (103). This finding has also been demonstrated in other mental health conditions, with early trauma exposure predicting poor response to treatment and longer time to recovery in individuals with major depressive disorder (104, 105). As such, current pharmacological treatment for individuals with psychosis may risk leaving traumatized individuals with an untreated and highly stressful environmental component, which is likely to continue to negatively impact both their neurochemistry and recovery. Complicating this matter, the experience of psychosis itself, and coercive elements of treatment such as involuntary hospitalization, have repeatedly been shown to induce trauma symptoms in up to two thirds of individuals experiencing psychosis, resulting in clinical PTSD in one in three such individuals (33–36).

Efforts to implement trauma screening in individuals with psychosis revealed that PTSD is drastically under-diagnosed in such populations (106). In a group of 2,608 individuals with a diagnosed psychotic condition, 0.5% had a previously identified PTSD diagnosis. After implementing trauma screening, this number increased to 16%, a striking 32-fold increase, demonstrating that 96.9% of PTSD cases in individuals with psychosis in this study had previously gone unreported and untreated.

### Psychosis and Treatment as Traumatic Experiences

**Figure 3:**
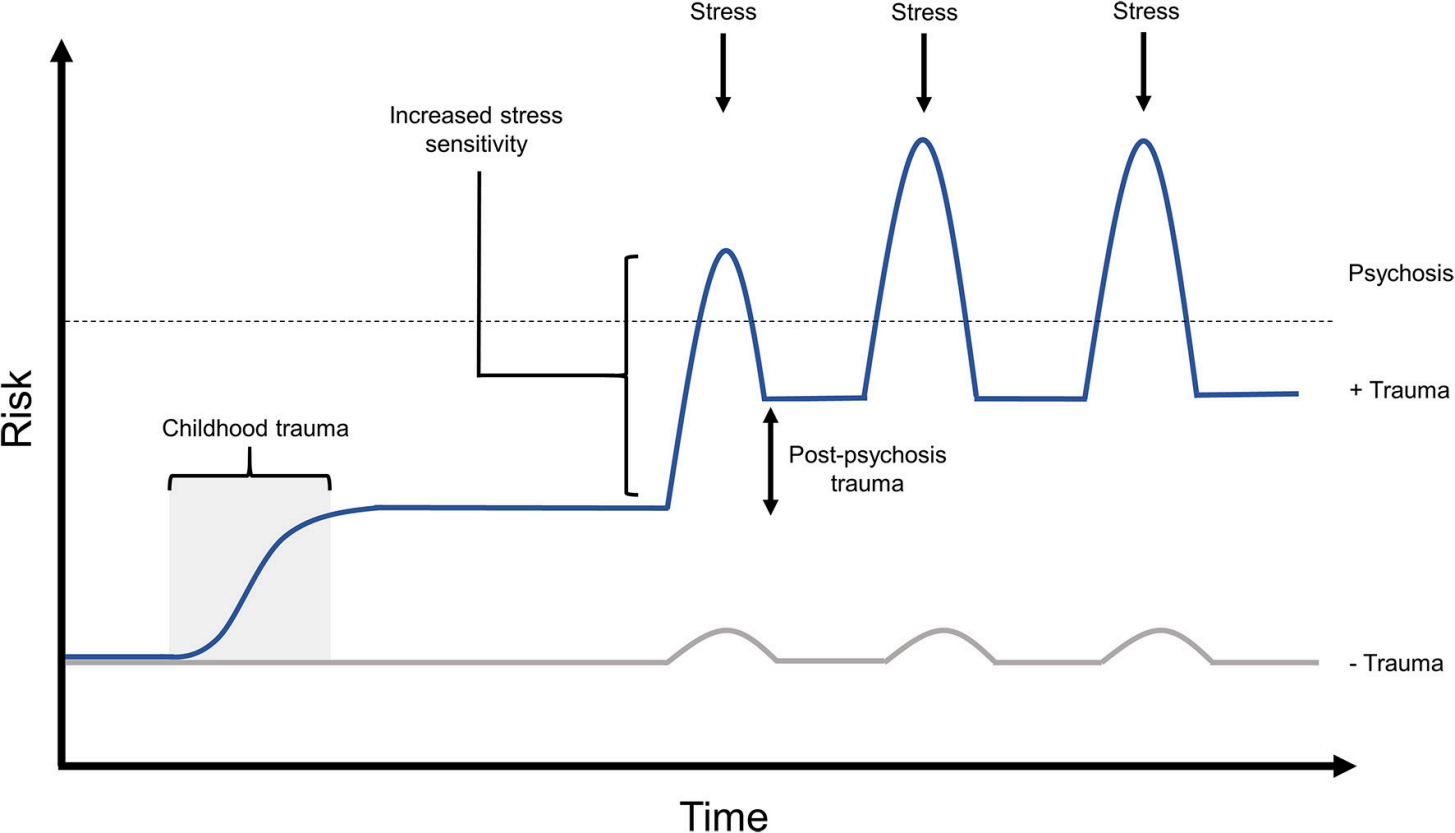
The proposed effects of post-psychosis trauma on relapse risk. Up to two thirds of individuals have been shown to experience post-psychosis trauma, the risk for which is significantly increased by exposure to prior trauma. It is likely that lingering post-psychosis trauma symptoms, if left untreated, would hinder an individual's ability to recover from this experience, and to maintain recovery when faced with later stressors. In this model, childhood trauma would significant raise an individual's baseline psychosis risk, while predisposing them to experience post-psychosis trauma. After an experience of psychosis, in this example triggered by a stressor, the individual's psychosis risk is further amplified by untreated post-psychosis trauma symptoms. This may result in a lower threshold for experiencing further episodes of psychosis in response to future stress, potentially also with the capacity for increased symptom frequency or intensity in later episodes.

A large proportion of individuals with experiences of psychosis go on to suffer clinical PTSD, or PTSD-like trauma symptoms from this experience, the risk for which is modulated by previous experience of childhood trauma (38). Studies have demonstrated that 66–69% of individuals meet the symptom criteria for PTSD with regard to psychosis- and treatment-related trauma, and 30–39% meet the full criteria for post-psychosis PTSD diagnosis (33–36). Exposure to childhood trauma has been shown to increase risk of experiencing post-psychosis trauma by 27-fold (38). Such work clearly demonstrates that experiences of childhood trauma not only predispose to experiences of psychosis, but are also likely to hinder recovery from psychotic episodes by leaving individuals vulnerable to experiencing post-psychosis trauma (Figure 3).

Further work with individuals that had experienced either a single episode, or multiple episodes, of psychosis also yielded similar results (34). In these studies, up to half of first episode and multiple episode individuals met the DSM-IV criteria for having experienced a traumatic event with regard to their experience of psychosis (33, 34). Similarly, almost a third of first episode and over a quarter of multiple episode individuals met the DSM-IV criteria for having experienced trauma in relation to the mental health treatment they received (33, 34).

A study of 395 individuals with a range of psychotic disorders found 69% of individuals reporting that one or more of their hospitalizations had been “traumatic or extremely distressing” (39). Of the individuals in this study reporting treatment-related trauma, the most frequently reported traumatic experiences were involuntary hospitalization (reported by 62%), being placed in physical restraints (40%), and being forcibly medicated (37%). Similarly, 73% of individuals reporting treatment-related trauma also reported feelings of intense helplessness, fear, and horror, with 42% believing that they could be seriously harmed or killed during treatment. Indeed, Paksarian et al. found that 39% of individuals reporting treatment-related distress met the DSM-IV criteria for having experienced a traumatic event (39).

Such studies would advocate for psychological therapy interventions, and particularly trauma-informed interventions, in supporting both those with psychosis influenced by prior trauma, and those in recovery from psychosis who may be prone to ongoing mental health difficulties or at risk of relapse due to lingering trauma symptoms from the experience of psychosis itself, and from any traumatic treatment-related events.

### Psychological Therapies for Individuals Experiencing Psychosis

Research into the utility of psychological interventions in psychosis have lagged behind that of other mental health conditions, such as depression and anxiety, likely due in part to the long-held view that psychosis and schizophrenia are largely biological conditions, or that individuals experiencing psychosis may struggle to fully engage with psychological therapy due to difficulties presented by ongoing symptoms (107). However, new research is beginning to demonstrate the safety and utility of psychological therapy for experiences of psychosis, particularly in the case of trauma-informed therapy and adaptations of cognitive behavioral therapy for psychosis.

Eye movement desensitization and reprocessing (EMDR), a form of therapy typically used to treat trauma and PTSD, has been shown to be safe and effective in significantly reducing trauma symptoms in individuals with diagnoses of concurrent psychosis and PTSD (48–50).

An assessment of the potential re-traumatizing effects of psychological intervention for individuals with trauma and psychosis compared symptom exacerbation, re-victimization, and adverse events in a single-blind randomized control trial comparing 108 individuals receiving trauma-focused treatment (TFT) with 47 individuals on a waiting list receiving treatment as usual (TAU) (49). In this study, TAU was administered in the form of multi-disciplinary care from the individual's community mental health team, typically involving antipsychotic medication, with further treatment and supportive counseling from multiple members of this team. In the treatment arm, 53 of the participants received prolonged exposure (PE) therapy, and 55 received EMDR. This treatment was delivered over the course of 10 weeks, in the form of 8 weekly sessions lasting 90 min each. As no significant difference in the outcomes was found between the PE and EMDR groups, the data was pooled to compare trauma-focused treatments vs. the waiting list condition. When comparing baseline to the post-treatment time point, those in the waiting list condition had significantly higher rates of self-reported PTSD symptom exacerbation compared to those receiving TFT, though this difference became non-significant at 6 months follow up. Similarly, those in the waiting list condition had significantly higher levels of general symptom exacerbation, and showed a trend toward reporting increased paranoid ideation post-treatment compared to those receiving TFT. No significant difference was found between the groups for clinician rated PTSD symptoms or depressive symptoms at the post-treatment time point. Those in the TFT group were found to be significantly less likely to experience adverse events during treatment and follow up, with a relative risk reduction of 27.9 and 34.6% post-treatment and at 6 months follow up, respectively. Similarly, the rates of re-victimization were significantly lower in the TFT group compared to waiting list at 6 months follow up, but not at the post-treatment time point (49). This work demonstrates that trauma-focused therapy is safe for use with individuals experiencing concurrent psychosis and PTSD, and is unlikely to cause distressing side effects or symptom exacerbation.

In a single-blind randomized control trial, 66 participants with FEP were randomly assigned to receive TAU or cognitive recovery intervention (CRI), a treatment aimed to reduce experiences of post-psychosis trauma (51). At 6 months follow up, those that had received CRI presented with significantly lower levels of post-psychosis trauma, with over two thirds reporting substantial improvement in trauma symptoms, compared to those receiving TAU, of which one third reported improvement. In particular, those reporting higher levels of pre-psychosis trauma benefited most from CRI (51), suggesting that individuals with prior trauma experiences may stand to benefit significantly from trauma-informed support. The authors note that failing to provide early cognitive therapy-based intervention to those experiencing first episode psychosis may put more than twice as many individuals at risk of lingering or worsening trauma symptoms following a psychotic episode (51).

In the UK, psychological therapy (particularly CBT) has been recommended as a frontline treatment for individuals both at risk of experiencing psychosis, and those experiencing psychosis (54). A recent single-blind randomized control trial involving 75 individuals compared the effects of antipsychotic medication alone, CBT alone, and a combination of antipsychotic treatment plus CBT (53). All treatment arms were found to significantly reduce symptoms, with total PANSS scores being significantly decreased in the combined treatment group compared to the CBT group alone. Reduction in PANSS scores was not significantly different between the treatment alone groups, or the antipsychotic and combined treatment groups. Morrison et al. found no difference in adverse events between the three groups, but reported fewer side effects in the CBT alone group (53). Such studies demonstrate the utility and feasibility of CBT as a treatment option for those with experiences of psychosis.

Similarly, an earlier single blind randomized control study in individuals not taking antipsychotic medications compared 74 individuals randomized to TAU or to TAU plus CBT (52). In this instance, TAU referred to regular and comprehensive care co-ordination and psychosocial interventions, with regular monitoring through PANSS assessment, as well as options for crisis management and family interventions. This trial further demonstrated the ability of CBT to significantly reduce psychiatric symptoms (as measured by PANSS score) in those receiving this intervention, compared to those receiving TAU, with the authors reporting an effect size comparable to those identified in meta-analyses of psychiatric medication efficacy. Change in symptoms included significant improvements in total PANSS score and scores for positive symptoms, as well as improvements in some dimensions of delusional beliefs, voice hearing, and social functioning. However, cognitive therapy intervention was not found to have an effect on negative symptoms in this study (52).

An additional trial involving 385 individuals receiving CBT demonstrated that this intervention was effective in bringing about significant and sustainable improvements in delusional beliefs, voice hearing, anxiety, depression, and quality of life, which were maintained at 6 months follow up compared to pre-therapy baselines (57). Similarly, a meta-analysis of randomized control trials comparing CBT vs. TAU in the treatment of delusions demonstrated that cognitive therapy intervention significantly improved delusional beliefs, an effect which was maintained to an average follow up time of 47 weeks across the studies (56). However, CBT was not found to be significantly different in terms of effectiveness in comparison to other psychological interventions. A further meta-analysis of 12 studies found that CBT resulted in long-term beneficial changes in positive symptoms in individuals whose condition had previously been identified as medication-resistant (55). As well as evidence demonstrating the effectiveness of CBT in improving positive symptoms, a recent meta-analysis by Lutgens et al. also provided evidence for CBT and a number of psychosocial interventions in improving negative symptoms of psychosis (108). Lutgens et al. assessed 95 studies reporting negative symptom outcomes in order to determine the effectiveness of CBT and psychosocial interventions on these symptoms (108). Of the 95 studies, 26 studies assessed the efficacy of CBT. In this analysis, CBT was found to be an effective intervention for the treatment of negative symptoms compared to TAU in 59% of studies. However, in 12 studies that used an active control, no significant difference was found between groups. This would suggest that other active psychosocial interventions may also be beneficial for individuals experiencing negative symptoms of psychosis. Indeed, Lutgens et al. also found evidence to support the utility of skills-, exercise-, and music-based interventions in the treatment of these symptoms (108).

Despite a number of positive meta-analyses such as those above (55, 56, 58, 109), debate around the efficacy of psychological therapy for psychosis continues (110). Some studies have failed to find beneficial effects for CBT in the treatment of psychosis (111), or found only small effects (112), while others have reported variable outcomes which are influenced by other factors, such as the relationship between the client and therapist (113). It is also important to understand the potential barriers that may prevent an individual from accessing or benefiting from such interventions. For example, a recent study by Hazell et al. found that both clients and clinicians felt that ongoing psychotic symptoms might be one of a number of challenges in implementing such interventions, along with additional practical barriers, such as lack of resources within mental health services (107). Further research in this area will be necessary to improve access to, and utility of, psychological therapy for those experiencing psychosis. Additional work by Hazell et al. has suggested that symptom-specific treatment may be one route to overcoming the identified barriers (114).

With regard to randomized control trials, it has been suggested that the variability inherent in tailoring psychological therapy to the needs of each client may make such interventions difficult to assess in a trial setting, leading to overgeneralized criteria that do not accurately assess the efficacy of therapy in achieving the client's individual goals (115). On this subject, some have criticized the use of outcome measurements which are often based on overall symptom scales, with the assertion that the most appropriate uses for CBT are in, for example, targeting specific dimensions of an individual's delusional beliefs, aiming to reduce distress, and aiming to improve the individual's ability to cope with such experiences (116). Birchwood et al. suggested that the intricacies of such changes were unlikely to be captured by generalized symptom measurements, despite the fact that they may represent significant benefits for the client. In support of this, qualitative research into service user priorities identified that managing stress and anxiety were among the most commonly endorsed immediate treatment priorities for those experiencing psychosis, with improved coping and emotional wellbeing identified as top long-term treatment priorities (117). Similarly, research addressing personal experiences of CBT demonstrated that coping strategies were among the most commonly cited key components of CBT for those receiving this intervention for psychosis (118). This would suggest that improved ability to cope with stressors is considered to be among the most useful and valuable benefits by clients, regardless of actual change in symptom frequency or severity. However, measures of such benefits are often not included when assessing the efficacy of therapy in clinical trials.

Despite such debate (the remainder of which is beyond the scope of this review), research is ongoing with the aim of resolving such questions, and further establishing the effectiveness of CBT for individuals with psychosis. Preliminary research is also being carried out to assess the suitability of a number of other therapeutic modalities for use with individuals experiencing psychosis, including Cognitive Analytic Therapy (CAT) (119, 120), Compassion Focused Therapy (CFT) (121, 122), and others, with the hope of providing additional choices with regard to evidence-based treatment options for those seeking psychological therapy-based care.

## Limitations

This work has a number of limitations that are of note. Firstly, this review aimed to cover a very wide range of topics from molecular biology to psychological therapy. Given the breadth of this article, there was insufficient space to present and deeply discuss the full range of supporting and opposing evidence for each topic. It did not systematically review the literature relating to control trials of psychological therapy for psychosis, nor did it evaluate the quality of evidence in the studies reported. While the authors endeavored to fairly and accurately represent the available literature, this lack of systematic procedure means that the introduction of bias cannot be ruled out. Secondly, this review covers only very specific and extreme experiences of early life stress and trauma. As discussed above, the studies referenced in this review focus almost exclusively on direct personal experiences of victimization, centered largely around interpersonal abuse and violence. It is important in this case to note that additional non- victimization-based, or non-direct experiences, such as sudden and life-threatening illness, or witnessing another person's experience of trauma, may also constitute traumatic experiences and have the potential to contribute to future mental health risk. Similarly, the effects of pre- and peri-natal stressors, such as maternal stress, were not included, nor were additional factors which may contribute to early life stress and psychosis risk, such as low socioeconomic status. While there was not scope in this review to do so, the authors believe it is also of import to acknowledge the intersecting effects of numerous personal and social categorizations (e.g., race, sexuality, gender identity, socioeconomic status) in influencing one's likelihood of experiencing discrimination, disadvantage, or victimization that may result in trauma or repeated exposure to stress, thereby contributing to risk of developing related psychiatric symptoms.

## Summary and Conclusions

Recent advances in the field of schizophrenia genetics have demonstrated the highly polygenic nature of this condition, with many common risk variants of low effect size spread widely across the non-coding genome (4, 13). That a significant number of identified risk variants fall within proposed regulatory regions (12, 15) would suggest that risk for schizophrenia or psychosis is likely to be modulated by environmental stressors in a genotype-dependent manner. Indeed, a significant number of schizophrenia GWAS SNPs have been shown to act as eQTLs or meQTLs, influencing gene expression or methylation in the human brain (69–71).

Childhood adversity is one of the largest preventable risk factors for experiencing psychosis (18, 19), and has been shown to significantly alter methylation across key stress response gene loci in humans (42–44). Exposure to childhood trauma has also been associated with alterations in brain structure and connectivity (91, 94, 95), with evidence to suggest that the effects of childhood trauma on brain structure can be influenced by genotype (101, 102). Such alterations in brain structure have been shown to be associated with increased stress reactivity (91), high levels of which is a known risk factor for psychosis (30), and has been shown to be associated with increased symptom severity in those experiencing psychosis or psychosis-like symptoms (32, 92). This traumagenic neurodevelopmental model of psychosis suggests that exposure to early life trauma can alter the trajectories of neurodevelopment and neurochemistry to influence response to future life stress in ways that would increase risk of experiencing psychosis (27, 29), and increase symptom severity in those with this condition (Figure 1). Given that over two thirds of people experience post-psychosis trauma symptoms (33, 34)—the likelihood of which is vastly increased by prior trauma exposure (38)—it is likely that lingering trauma symptoms and heightened stress sensitivity (32) will negatively impact the individual's ability to achieve or maintain recovery in the face of day-to-day or future stressors (Figure 3). Treating only with pharmacological interventions is unlikely to resolve such distress, or equip the individual with useful coping strategies, and as such, repeated experiences of stress will continue to act on the individual's mental health in a G × E manner.

**Figure 4:**
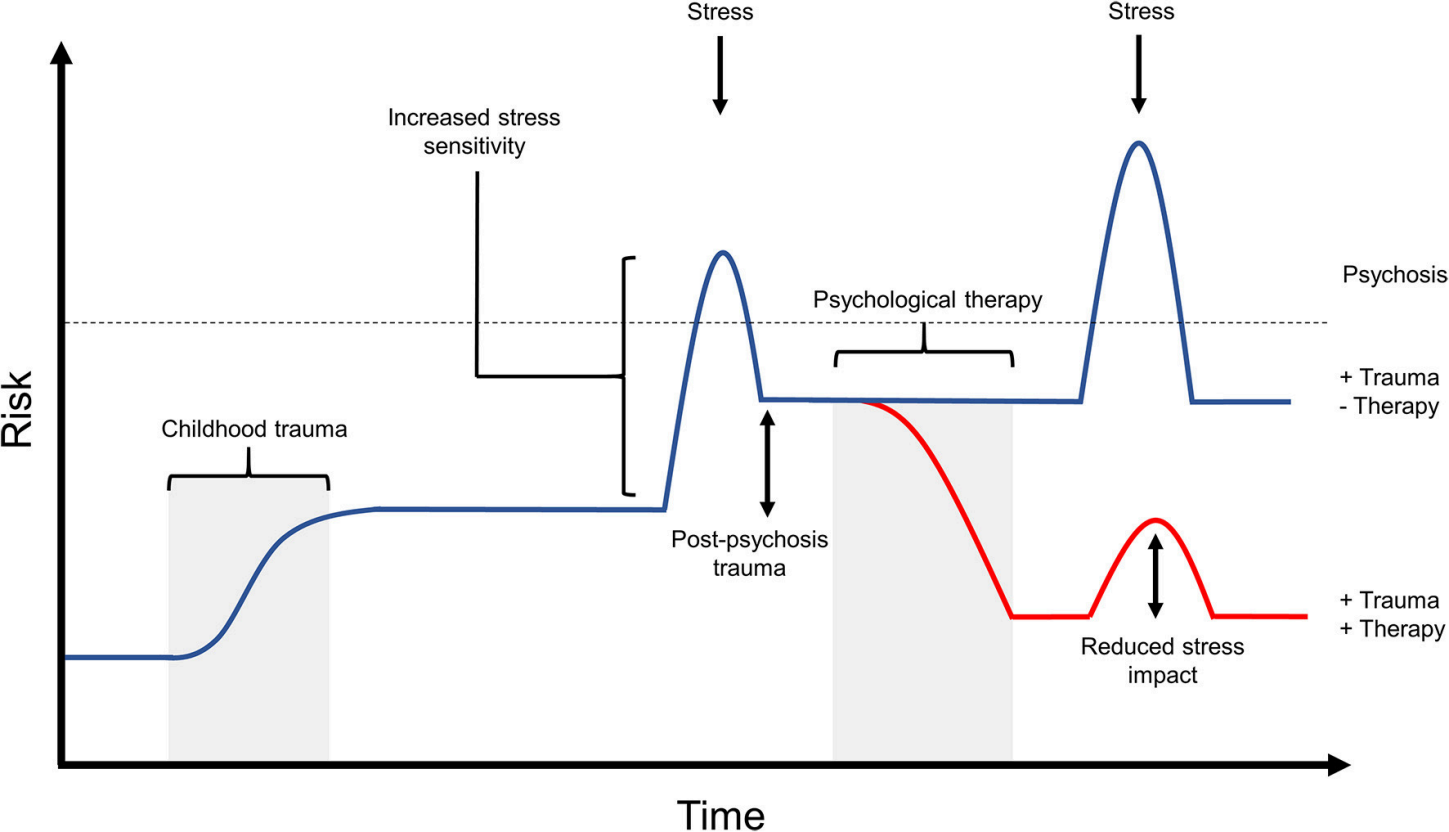
The potential effects of psychological therapy on post-psychosis trauma and stress impact. Recent research into psychological therapy for psychosis is beginning to demonstrate the ability of such interventions to improve both psychosis symptoms as well as secondary issues in those experiencing psychosis, such as anxiety and depression. Similarly, psychological therapy is able to reduce distress associated with psychosis symptoms, and improves the individual's ability to cope with stressors. This diagram outlines one potential risk trajectory that may be associated with effective psychological therapy intervention. In this example, the individual has experienced early life trauma and post-psychosis trauma, which resulted in very high risk for experiencing relapse in response to further stress. If this individual receives successful therapy after their first psychotic episode, this could decrease risk of further episodes by working to resolve both early and recent trauma experiences, and by providing the individual with an improved ability to cope with stress. As can be seen in this figure, subsequent stress following therapy may have a smaller effect on risk due to the individual's improved capacity for coping. While not shown here, psychological therapy intervention may also be provided as a preventative measure to those at high risk for psychosis. Such intervention would similarly aim to improve coping and reduce the effect of stress, with the hope of preventing a psychotic episode in the future.

Thinking in this G × E way about psychosis would advocate for the role of psychological therapies or other social support as ways to help a person cope with or resolve the stress in their life, that may be preventing them from achieving or maintaining recovery (Figure 4). Recent studies into the utility and effectiveness of psychological therapy for psychosis have demonstrated that trauma-informed therapies are safe and effective for use with such clients (48–50), and can reduce rates of re-victimization and post-psychosis trauma (49, 51). Similarly, meta-analyses assessing the effectiveness of CBT for psychosis have demonstrated that such interventions can provide sustainable improvements in positive symptoms (56, 58, 109), including in individuals that had previously been classified as “treatment resistant” (55).

A wealth of research, both biological and psychological, has demonstrated the effects of stressful life experiences on our epigenetics and subsequent risk of developing a mental health condition, though research into the epigenetic effects of positive experiences and their effect on mental health are less studied. Given the genetic data showing that common schizophrenia risk falls largely in regulatory regions which are likely to be responsive to environmental challenge or change, support that positively affects an individual's day-to-day environment or stress levels may be a feasible route to positive and therapeutic epigenetic change. Indeed, preliminary studies into the biological effects of therapy have demonstrated that psychological interventions can modulate biological mechanisms relevant to mental health, such as altering methylation over key CNS genes (45–47, 123), and modulating connectivity between brain regions that are relevant to psychosis in ways which correlate with clinical improvement in symptoms (124, 125). While further research is needed to address a number of questions and criticisms around the use of psychological therapy for psychosis, such interventions provide hope, particularly for those who find antipsychotic treatment unhelpful or intolerable, and for those with experiences of trauma, the ongoing effects of which are unlikely to have been adequately appreciated or addressed in regard to their mental health.

## Author Contributions

OG wrote the draft manuscript. VB and JQ provided feedback for revisions. All authors contributed to and approved the final manuscript.

### Conflict of Interest Statement

The authors declare that the research was conducted in the absence of any commercial or financial relationships that could be construed as a potential conflict of interest.
